# Product Manifold Representations for Learning on Biological Pathways

**Published:** 2025-02-04

**Authors:** Daniel McNeela, Frederic Sala, Anthony Gitter

**Affiliations:** 1 Department of Computer Sciences, University of Wisconsin-Madison, Madison, WI, USA; 2 Morgridge Institute for Research, Madison, WI, USA; 3 Department of Biostatistics and Medical Informatics, University of Wisconsin-Madison, Madison, WI, USA.

## Abstract

Machine learning models that embed graphs in non-Euclidean spaces have shown substantial benefits in a variety of contexts, but their application has not been studied extensively in the biological domain, particularly with respect to biological pathway graphs. Such graphs exhibit a variety of complex network structures, presenting challenges to existing embedding approaches. Learning high-quality embeddings for biological pathway graphs is important for researchers looking to understand the underpinnings of disease and train high-quality predictive models on these networks. In this work, we investigate the effects of embedding pathway graphs in non-Euclidean mixed-curvature spaces and compare against traditional Euclidean graph representation learning models. We then train a supervised model using the learned node embeddings to predict missing protein-protein interactions in pathway graphs. We find large reductions in distortion and boosts on in-distribution edge prediction performance as a result of using mixed-curvature embeddings and their corresponding graph neural network models. However, we find that mixed-curvature representations underperform existing baselines on out-of-distribution edge prediction performance suggesting that these representations may overfit to the training graph topology. We provide our Mixed-Curvature Product Graph Convolutional Network code at https://github.com/mcneela/Mixed-Curvature-GCN and our pathway analysis code at https://github.com/mcneela/Mixed-Curvature-Pathways.

## 1. Introduction

Machine learning methods for embedding graphs (Wu et al., 2021) enable learning on data ranging from social media networks, to proteins and molecules, to phylogenies and knowledge graphs. These embeddings then enable useful node classification and edge prediction models, which can perform tasks as diverse as predicting whether a molecule is active against a given drug target or whether two users are likely to share a preference for a given product.

While traditional graph learning methods employ Euclidean representations (Grover & Leskovec, 2016), it is known that for certain graphs Euclidean representations are unable to perfectly preserve graph structure, regardless of what algorithm is used (Bourgain, 1985). As a result, recent works have studied whether lower graph distortion can be achieved by embedding in non-Euclidean spaces, such as hyperbolic space (Sala et al., 2018). Generally speaking, lower distortion correlates with better downstream task performance.

The works of Gu et al. (2019) and Giovanni et al. (2022) have examined embeddings into mixed-curvature products of spaces and heterogeneous manifolds, respectively. By “mixed-curvature products of spaces”, we mean a Cartesian product of embedding spaces having constant positive, negative, or zero curvature. These correspond to the model spherical, hyperbolic, and Euclidean spaces, respectively (Lee, 2018). While these methods have been evaluated on standard graph benchmarking datasets, their hypotheses have not been validated for specialized graphs found in biological pathways and networks, which may have properties and topologies that differ from general graphs in other domains. The product space approach is appealing because deciding a priori which space to embed into can be challenging.

*Biological pathways* are graphs that encode cellular processes. Typically, the nodes in such graphs are entities such as genes, proteins, or metabolites, and the edges designate relationships between them. For example, an edge connecting nodes A and B might indicate that the presence of protein A controls the transcription of gene B. Pathways are an important object of study in network biology as they can be used to infer subcellular relationships and understand the mechanisms underlying disease.

Embedding biological pathways is difficult because no canonical methods exist. Pathways exhibit a high degree of complexity. Some, due to a lack of study, are sparse while others exhibit high inter-connectivity. Their complex network structures suggest that non-Euclidean representations might provide significant benefits. However, no systematic study of embedding methods applied to biological pathway graphs has been undertaken. Only Euclidean embedding methods have been applied to pathway graphs (M A Basher & Hallam, 2020; Pershad et al., 2020), and because pathway graphs differ from standard graphs used to benchmark non-Euclidean embedding models, it is unknown to what extent these models would work for pathway graphs.

In this work, we study non-Euclidean embeddings of biological pathway graphs and their performance relative to standard Euclidean embeddings.^1^ We perform a large-scale test of a variety of embedding methods to pathway graphs taken from PathBank, Reactome, HumanCyc, the NCI Pathway Interaction Database, and KEGG and embed into a number of different combinations of spaces. For each pathway graph, we determine a best embedding space, as measured by the lowest graph distortion, and learn the node embeddings for that graph. Although biological pathway databases are of high quality, they are incomplete and only capture a fraction of the knowledge about the relevant biological processes (Hanspers et al., 2020). Therefore, we investigate the downstream performance of our graph embeddings by predicting potentially missing pathway edges. Instead of only predicting held out pathway edges, we also examine the considerably more challenging task of predicting protein-protein interactions (PPIs) from an independent database that may participate in the pathway. We find that mixed-curvature representations outperform, as measured by distortion, Euclidean representations in all cases. Furthermore, the positive impact of improved embeddings seems to generalize to downstream tasks such as edge prediction, where we find improvements in area under the receiver operating characteristic curve (AUROC) and average precision (AP) for held out in-distribution edges. However, for out-of-distribution STRING PPI edges, we find that the mixed-curvature representations underperform baselines, perhaps suggesting that these representations overfit to the in-distribution graph topology.

## 2. Background and Related Work

### 2.1. Non-Euclidean Embeddings and Machine Learning

Much of the research into non-Euclidean embeddings in machine learning originated in studies of graphs and networks, where they were originally used to embed concept ontologies. For example, Nickel & Kiela (2017) developed a method for embedding into the Poincaré model of hyperbolic space and used it to generate node embeddings for the WORDNET ontology. Sala et al. (2018) expanded on this work by determining the precision-dimensionality tradeoffs inherent in hyperbolic embeddings. Ganea et al. (2018) and Chami et al. (2019) then produced generalized Hyperbolic Neural Networks and Hyperbolic Graph Convolutional Networks (GCNs) to perform prediction directly in hyperbolic space on data of various types. Gu et al. (2019) extended graph representation learning to a Cartesian product of hyperbolic, spherical, and Euclidean spaces, while Giovanni et al. (2022) further generalized this to a product of manifolds of heterogeneous curvature. Our approach follows that of Gu et al. (2019). More recently, hyperbolic and non-Euclidean geometries have been applied to contrastive learning (Desai et al., 2023) and attention blocks (Tseng et al., 2023).

Hyperbolic modeling approaches have been applied to embed biological and chemical data exhibiting hierarchical structure. Many methods focus on drug discovery, specifically drug repurposing (Lau et al., 2023) or drug target prediction (Yue et al., 2023; Poleksic, 2023; Zahra et al., 2023; Pogány & Antal, 2024). The hierarchical relationships in the Gene Ontology make it appealing for learning hyperbolic representations of its terms and their associated genes (Kim et al., 2021; Li et al., 2023). In transcriptomics, methods have been developed to visualize and estimate the curvature of gene expression samples (Zhou & Sharpee, 2021), embed single-cell RNA-seq data (Klimovskaia et al., 2020; Ding & Regev, 2021), and model differential expression signatures (Pogány & Antal , 2023). Finally, embedding biological sequences has enabled visualizing members of a protein family (Susmelj et al., 2023) and Bayesian phylogenetic inference (Macaulay et al., 2023).

### 2.2. Pathway Graphs and Embeddings

Pathway graphs have been well-studied from a biological perspective, but embedding them to facilitate downstream prediction tasks is relatively new. For example, M A Basher & Hallam (2020) developed a method called pathway2vec, which combines five different Euclidean embedding methods to learn embeddings of biological pathways. Similarly, Pershad et al. (2020) used node2vec to generate embeddings of PPI networks and used the resulting embeddings as one component of a method to predict response to psychiatric drugs. Pathway embeddings are a crucial input to models that operate on pathway network structures to make predictions. Euclidean GCNs have been broadly applied to biological pathways to predict cancer subtypes (Lee et al., 2020; Hayakawa et al., 2022), synthetic lethality (Lai et al., 2021), PPIs (Pham & Dang, 2021), cancer survival (Liang et al., 2022), textual pathway descriptions (Yang et al., 2022), and subcellular localization (Magnano & Gitter, 2023). However, there has been no systematic study investigating the use of non-Euclidean and mixed-curvature embedding models for pathway graphs.

### 2.3. PPI Prediction

PPIs can be predicted based on combinations of proteins’ sequence, expression, functional, evolutionary, or 3D structural features (Durham et al., 2023). One PPI prediction formulation is as an edge or link prediction task in a network of known PPIs (Li et al., 2022). This can be accomplished using features from the original graph or by learning node embeddings as features for the edge prediction task. For example, Feng et al. (2020) predict signaling cascades from PPI graphs by integrating transcriptomics and copy-number data into a GCN. Jiang et al. (2020) use graph embeddings to predict links that indicate an enzymatic reaction between pairs of molecules from the KEGG database. Finally, Zhang & Kabuka (2019) and Liu et al. (2020) leverage a combination of sequence and network information to predict PPIs.

## 3. Methods

### 3.1. Data Processing

We downloaded five pathway datasets from Pathway Commons (Rodchenkov et al., 2020) v12, namely, PathBank (Wishart et al., 2020), Reactome (Gillespie et al., 2022), HumanCyc (Romero et al., 2004), KEGG (Kanehisa et al., 2012), and the NCI Pathway Interaction Database (Schaefer et al., 2009). We used the .txt files containing interaction participants, edge types, and associated metadata. For each pathway in each dataset, we created a NetworkX (Hagberg et al., 2008) graph object and ignored Pathway Commons edge types, treating each edge as undirected with no additional annotations. We then generated edge lists for the undirected graphs and learned embeddings using the mixed-curvature embedding Python library (Gu et al., 2019). All subsequent experiments were performed only for those pathway graphs for which a unique “best” embedding space could be determined, defined as the space exhibiting minimum distortion. Those pathways which did not exhibit a unique embedding space of minimum distortion were discarded as there exists no canonical way to select a representative product space to compare embedding and edge prediction methods against. We also discarded one pathway from the Reactome dataset having 36,293 edges because its runtime on the edge prediction task was prohibitive and it is an extreme outlier in terms of pathway size (Table 1).

For edge prediction, we downloaded Homo sapiens PPIs from STRING v12.0 (Szklarczyk et al., 2023). In STRING, PPIs are listed as pairs of ENSEMBL protein identifiers. For each gene or protein node name in the given pathway dataset, we map it to its UniProt SwissProt identifier using the *My-Gene* API (Xin et al., 2016). For each ENSEMBL protein identifier, we map it to the Ensembl_HGNC_uniprotid listed in the STRING aliases file. We then iterate over all STRING PPI edges. For each pathway, if there exists a STRING PPI corresponding to two UniProt identifiers in the given pathway, we add an edge to the potential test set for that graph. We only included STRING edges with experimental evidence and discarded all edges with a score of less than 500 out of 1000. This yields a dataset of reasonable size for edge prediction, although the score used to filter edges can be tuned. Filtering using higher experimental evidence scores would likely yield less false positives in the list of candidate edges for each pathway graph; however, the relationship here is not strictly linear.

### 3.2. Learning Embeddings

We adopt the notation ${\text{S}}_{C}^{s_{i}}$ for the spherical space having constant positive curvature C and dimension $s_{i},{\text{H}}_{C}^{h_{j}}$ for the hyperbolic space having constant negative curvature -C and dimension $h_{j}$, and ${\text{E}}^{e_{k}}$ for the Euclidean space having zero curvature and dimension $e_{k}$. Given a collection of spherical, hyperbolic, and Euclidean spaces, each having an associated dimension and curvature, we write their Cartesian product space as

(1) $$𝒫=\prod_{i=1}^{m}{\text{S}}_{C_{i}}^{s_{i}}\times \prod_{j=1}^{n}{\text{H}}_{C_{m+j}}^{h_{j}}\times \prod_{k=1}^{p}{\text{E}}^{e_{k}},$$

Here the product space 𝒫 consists of m+n+p spaces and has total dimension $\sum_{i}s_{i}+\sum_{j}h_{j}+\sum_{k}e_{k}$. Following Gu et al. (2019), we call each individual ${\text{S}}^{s_{i}},{\text{H}}^{h_{i}},{\text{E}}^{e_{i}}$ a component and the decomposition of the product space into components as in Eq. 1 the signature of the space.

We learn an embedding function f:𝒢→𝒫 where 𝒢 is the space of pathway graphs and 𝒫 is a product manifold with a fixed, predefined signature. For embedding node u of a graph G on a product manifold 𝒫 having kth hyperbolic component ${\text{H}}_{C_{j}}^{h_{j}}$ with curvature $-C_{j}$, we randomly initialize $f^{k}(u)=(p_{0},p_{1},\ldots ,p_{h_{j}})$ to a point on the hyperbolic manifold, parameterized using the hyperboloid model, i.e.

(2) $$-{p_{0}}^{2}+{p_{1}}^{2}+\ldots +{{p_{h}}_{j}}^{2}=-C_{j},$$

Similarly, for the lth spherical component with curvature $C_{i}$, we randomly initialize $f^{l}(u)=(p_{0},p_{1},\ldots ,p_{s_{i}})$ lying on the sphere

(3) $${p_{0}}^{2}+{p_{1}}^{2}+\cdots +{{p_{s}}_{i}}^{2}=C_{i}$$

and for the mth Euclidean component, we randomly initialize $f^{m}(u)=(p_{1}+\cdots p_{e_{k}})$. For the Euclidean components, f is unconstrained. For the hyperbolic and spherical components, we require one extra dimension because we embed hyperbolic and spherical spaces of dimension n in ${\text{R}}^{n+1}$. Squared distances in the product space decompose as sums of squared distances in the component spaces (Lee, 2018), i.e.

(4) $$d_{𝒫}(u,v{)}^{2}=\sum_{i}d_{M_{i}}{\left(\pi_{i}(u),\pi_{i}(v)\right)}^{2}$$

for u,v∈𝒫, where $\pi_{i}$ denotes projection onto the ith component. The learning of f takes place via optimization of the following loss function

(5) $$𝓛(f)=\sum_{1\leq u<v\leq n}\left|{\left(\frac{d_{𝒫}(f(u),f(v))}{d_{G}(u,v)}\right)}^{2}-1\right|$$

where $d_{G}(u,v)$ denotes the graph distance between nodes u and v, defined as the length of the shortest path connecting them in G, and $d_{𝒫}(f(u),f(v))$ denotes the product manifold geodesic distance between the learned embeddings for nodes u and v. To learn the embedding function, we initially randomize the embeddings for all nodes, then train f so as to minimize 𝓛 using the Riemannian stochastic gradient descent optimization algorithm (Bonnabel, 2013), which extends stochastic gradient descent to arbitrary Riemannian manifolds.

The main metric used to evaluate our embeddings is the average graph distance distortion. We define the distortion 𝒟 of a learned embedding f relative to a graph G=(V,E) to be

$$𝒟(f)=\frac{1}{|V{|}^{2}}\sum_{u,v\in V,u\neq v}\frac{\left|d_{𝒫}(f(u),f(v))-d_{G}(u,v)\right|}{d_{G}(u,v)}$$

### 3.3. Hyperparameters

We create 252 embeddings for each pathway graph corresponding to different combinations of the number of hyperbolic, spherical, and Euclidean spaces; the learning rate; and the dimensionalities of each space (Appendix). We test having different numbers of components of each space, where the number of components of each type ranges from 0 to 3. We also constrain the total dimensionality of all spaces to sum to 100. This is to keep the comparison across different combinations fair by ensuring they each have the same representational capacity as governed by the number of dimensions.

### 3.4. Mixed-Curvature Product GCN

Similar to Bachmann et al. (2020), we extend the Hyperbolic GCN of Chami et al. (2019) to products of constant curvature manifolds. The following facts of Riemannian geometry allow us to extend GCNs from single manifolds to product manifolds.

#### Definition 3.1.

Given Riemannian manifolds, $\left(M_{1},g_{1}\right),\ldots ,\left(M_{n},g_{n}\right)$, we can construct a Riemannian product manifold $\left(M_{1}\times M_{2}\times \cdots \times M_{n},g\right)$ with the product metric, $g=g_{1}⊕\cdots ⊕g_{n}$, where the distance decomposes as

(7) $$d_{M}(x,y{)}^{2}=d_{M_{1}}{\left(x_{1},y_{1}\right)}^{2}+\cdots +d_{M_{n}}{\left(x_{n},y_{n}\right)}^{2}$$

for any $x=\left(x_{1},\ldots ,x_{n}\right),y=\left(y_{1},\ldots ,y_{n}\right)\in M$.

Thus, operations in the tangent spaces, $T_{p}M$, of the product manifold M decompose as direct sums of operations over the constituent tangent spaces $T_{p_{i}}M_{i}$, where $M=M_{1}\times \cdots \times M_{n}$ and $p=\left(p_{1},\ldots ,p_{n}\right)$.

On each pathway graph for which we perform edge prediction, we match the signature of the product manifold in the GCN to the one used to learn the pathway embedding. The operations in the Product GCN are defined analogously to the ones in the hyperbolic GCN paper (Chami et al., 2019). Briefly put, pathway nodes are represented as a Cartesian product of points on some combination of components of ${\text{H}}^{n},{\text{S}}^{n}$, and ${\text{E}}^{n}$ manifolds. At each layer in the Product GCN, the points are projected into a tangent space to the manifold via the log map of the product space and a GCN operates in this Euclidean tangent space using the standard message-passing updates. The points are then projected back onto the manifold after the layer operation via the product space exp map. Both the log map and the exp map decompose as sums over the constituent manifolds in the product space. The same procedure is used for attention and activation layers, with projection into the tangent space, followed by a standard layer operation, followed by projection back onto the manifold. For spherical (S) and hyperbolic (H) manifolds having curvatures C and −C, respectively, the exp and log maps are defined as follows:

$$(\text{S})\;{\text{exp}}_{\text{x}}^{C}\left(\text{v}\right)=\text{cos}\left(\frac{\text{v}}{\left\|\text{v}\right\|}\right)\text{x}+C\text{sin}\left(\frac{\text{v}}{\left\|\text{v}\right\|}\right)\text{v}$$

$$(\text{H})\;{\text{exp}}_{\text{x}}^{C}\left(\text{v}\right)=\text{cosh}\left(\frac{\left\|\text{v}\right\|}{\sqrt{C}}\right)\text{x}+\sqrt{C}\;\text{sinh}\left(\frac{\left\|\text{v}\right\|}{\sqrt{C}}\right)\frac{\text{v}}{\left\|\text{v}\right\|},$$

$$(\text{S})\;{\text{log}}_{\text{x}}^{C}(\text{y})={\text{cos}}^{-1}\left(\langle \text{x},\text{y}\rangle_{C}\right)\frac{\text{y}-\text{x}-\langle \text{x},\text{y}-\text{x}\rangle_{C}}{\left\|\text{y}-\text{x}-\langle \text{x},\text{y}-\text{x}\rangle_{C}\right\|}$$

$$(\text{H})\;{\text{log}}_{\text{x}}^{C}(\text{y})=\sqrt{C}\;\text{arcosh}(-\langle \text{x},\text{y}\rangle /C)\frac{\text{y}+\frac{1}{C}\langle \text{x},\text{y}\rangle \text{x}}{\left\|\text{y}+\frac{1}{C}\langle \text{x},\text{y}\rangle \text{x}\right\|}$$

The Product GCN is trained using the Riemannian Adam optimization algorithm (Becigneul & Ganea, 2019) on the edge prediction task described in Section 4.2.

## 4. Results

### 4.1. Pathway Embeddings

**Figure 1.**
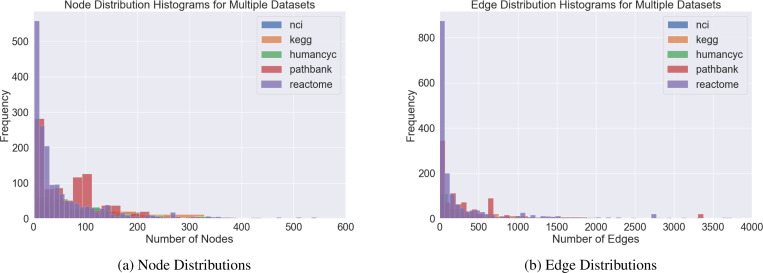
Histograms of node and edge distributions for the pathway databases studied. A few outliers were excluded.

We first summarize each of the pathway databases in Table 1 and provide node and edge histograms in Figure 1. There exist uniquely beneficial product spaces, as measured by distortion, for only roughly half of the pathways. Most pathways are small by graph machine learning standards with the majority having less than 100 nodes and less than 200 edges. Thus, pathways exhibit a unique opportunity to study the effects of popular graph learning techniques, including GCNs, on small graphs, as most graph machine learning benchmarks possess thousands, or even millions and billions, of nodes and edges.

**Figure 2.**
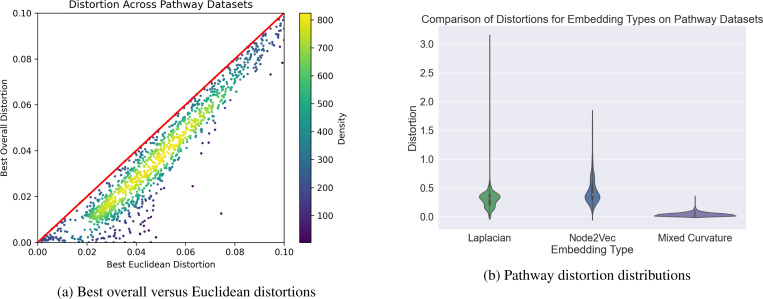
Best overall mixed-curvature versus best Euclidean distortions across all pathway datasets (a). See Figure 4 for individual pathway datasets. Distortions across all datasets for the graph Laplacian, node2vec, and mixed-curvature embedding methods (b).

Next, for each pathway graph, we determine the best combination of hyperbolic, Euclidean, and spherical components as determined by lowest distortion. Figure 2(a) demonstrates the reductions in distortion gained from learning the non-Euclidean embeddings over all graphs in each of the five pathway datasets we studied. The red diagonal indicates the position at which the distortions of the Euclidean embeddings and mixed-curvature product embeddings would be equal. Points lying below this line indicate graphs for which a product representation yielded a reduction in distortion over a Euclidean representation. Because a fully Euclidean embedding is a special case of the mixed-curvature product embedding, the best mixed-curvature product embedding should always have better distortion than the best Euclidean embedding. The question is whether the improvement is marginal or substantial on biological pathways. We observe that mixed-curvature product spaces provide marked reductions in distortion relative to the standard Euclidean embedding, with many graphs achieving a greater than 50% reduction in distortion.

We further compare our mixed-curvature embeddings against two common graph embedding baselines. The first is node2vec (Grover & Leskovec, 2016), a popular graph embedding method which uses an algorithm analogous to word2vec (Mikolov et al., 2013) trained on random walks taken from the graph. We also compare against a standard embedding method formed by taking eigenvectors of the graph Laplacian matrix (Bonald, 2019). For both methods, we embed the pathway graphs from all datasets and compute distortions for each pathway embedding in each dataset. Because node2vec and the graph Laplacian embedding do not directly minimize distortion in their loss functions, they can arbitrarily scale embedding vectors, causing them to perform poorly on a naive calculation of the distortion metric. Therefore, we perform a scaling optimization for the node2vec and graph Laplacian embeddings, finding for each dataset and embedding type the constant c which minimizes the below objective

$$\underset{c\in {\text{R}}^{+}}{\text{min}}\sum_{i<j}\left(\frac{c\cdot d\left(x_{i},x_{j}\right)}{d_{G}\left(n_{i},n_{j}\right)}-1\right)$$

where $d\left(x_{i},x_{j}\right)$ is the distance between the embeddings of nodes i and j, and $d_{G}\left(n_{i},n_{j}\right)$ is the shortest-path graph distance between nodes i and j. After calculating the optimal scaling factor, c, we scale the embeddings by this factor and recalculate the distortions for all pathways in the dataset. We summarize the distortions in Figure 2(b). The mixed-curvature embeddings exhibit significantly lower mean distortion and variance than the node2vec and Laplacian baselines.

### 4.2. Edge Prediction

#### 4.2.1. OVERVIEW

We train on individual pathway graphs to predict a set of held-out edges, then test our prediction models on the test set of experimentally validated edges from STRING. For each pathway, we determine its optimal embedding space using the results of our hyperparameter sweep for the distortion task. That is, for each pathway graph, we select the signature for the embedding space (number of hyperbolic, Euclidean, and spherical components, as well as their respective dimensionalities) that minimizes the distortion across the set of embeddings for that pathway graph. We then perform edge prediction on two different datasets:

##### In-Distribution

An in-distribution validation set containing held-out edges from the original pathway graph. These edges are not seen by the model during training, but the validation set performance metrics, such as validation accuracy and validation loss, *are* used to guide model training and hyperparameter selection. 95% of the edges from each pathway were used for training, and 5% were used for validation.

##### Out-of-Distribution

An out-of-distribution test set that includes edges from the STRING PPI database. This dataset is not seen by the model during training and *is not* used to guide model training or hyperparameter selection. These edges are general PPIs and do not necessarily have the same biological context as the pathway. However, pathway edge predictions supported by a general PPI are more plausible than those that are not.

We expect that the Product GCN, initialized with the mindistortion product space embeddings, has a better inductive bias in its pretrained representations that more preferentially match the topology of each graph than any of the other baselines. Thus, we expect that the Product GCN should more accurately predict in-distribution edges held out from the original graph. Although we are less certain what the effect of introducing non-Euclidean geometry should be to the prediction of out-of-distribution edges from an external database, we would hope that the Product GCN would still outperform the other baselines on this task.

#### 4.2.2. BASELINES AND PRODUCT GCN

We compare four different methods on the edge prediction task:

1. A Euclidean GCN initialized with pretrained node2vec embeddings.
2. A Euclidean GCN initialized with pretrained Laplacian embeddings.
3. A Euclidean GCN initialized with pretrained Euclidean embeddings designed to minimize distortion in ${\text{R}}^{n}$.
4. A Product GCN architecture that matches the signature of the optimal non-Euclidean pathway embedding as measured by minimum distortion and is initialized with the pretrained mixed-curvature embeddings from the first task described in Section 4.1.

The pretrained Euclidean embeddings in method 3 are learned using the same Python package as is used for learning the mixed-curvature embeddings (Gu et al., 2019) but use a Euclidean manifold as the target output space. For all methods, we keep the total dimensionality of the embedding space fixed at 100 dimensions, with the exception of the Laplacian embedding method for which the embedding dimension was set to be the number of nodes in the graph, |V|. Since most pathway graphs have |V|≤100 (Figure 1(a)) the reduced dimensionality of the Laplacian embedding would be expected to be harmful, though it could provide an outsized benefit in representational capacity for pathway graphs with a large number of nodes.

For the Product GCN, we match the signature of the best mixed-curvature embedding. For example, if the best embedding for pathway 21 of the PathBank dataset has the signature ${\text{H}}^{2}\times {\text{S}}^{3}\times {\text{E}}^{2}$, then we use a Product GCN architecture with two hyperbolic manifold layers, three spherical manifold layers, and two Euclidean manifold layers. We furthermore split each node embedding into its constituent pieces as determined by the product manifold signature. In this example, the embedding would be split into 2+3+2 = 7 slices, each of dimension 14. Each slice of the embedding is fed into the GCN for its corresponding manifold. Scores are then generated by each of the component GCNs and averaged to produce a final output score that is used to predict the presence of an edge between a given pair of nodes.

#### 4.2.3. MODEL TRAINING

For each graph on which we perform edge prediction with the Product GCN, we perform a sweep over 324 hyperparameter combinations (Appendix) and choose the one with the highest average of validation set AUROC and AP for use in downstream analyses. For the baseline methods, we perform a sweep over 108 hyperparameter configurations (Appendix). The difference in the two sweeps arises from the fact that the Product GCN has one additional hyperparameter, the curvature of the hyperbolic and spherical embedding spaces. We sweep over three values for this parameter, yielding three times as many hyperparameter configurations.

#### 4.2.4. SUMMARY OF RESULTS

**Figure 3.**
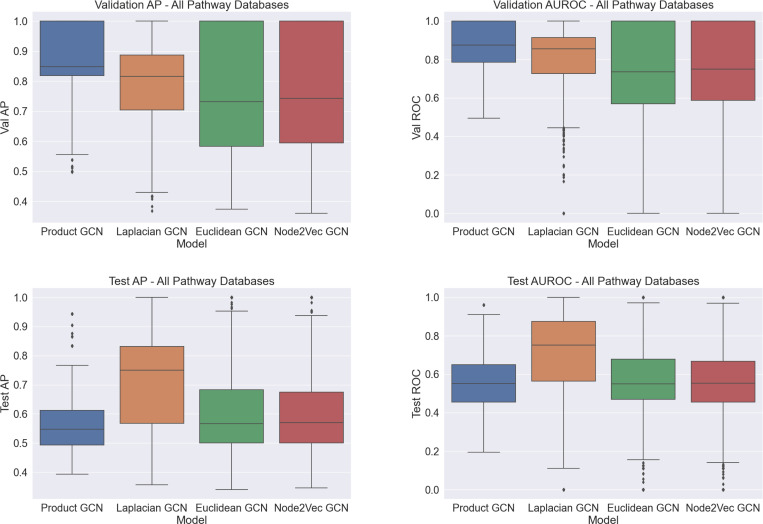
Edge prediction performance on all five pathway datasets for all models as given by four metrics: Validation Set AP, Validation Set AUROC, Test Set AP, and Test Set AUROC. Test set edges come from STRING.

We find that mixed-curvature product manifold representations yield substantial benefits in representational capacity for boosting downstream edge performance on the in-distribution validation edges (Figure 3). The Product GCN initialized with pretrained product manifold representations outperforms, on average, all baselines for prediction of in-distribution validation set edges. Among the baseline methods, the graph Laplacian-based Euclidean GCN outperforms the two other Euclidean GCNs. For pathways with more than 100 nodes, this advantage is likely due to its larger embedding dimension. In the Appendix, we provide detailed paired comparisons of the AP and AUROC for the Product GCN and each Euclidean GCN on individual graphs in each pathway database.

The Product GCN does not have the same advantage over the baselines for the prediction of out-of-distribution PPI edges from STRING. The Euclidean GCN with pretrained graph Laplacian embeddings is the clear best model, and all others have near random AUROC. We surmise that the introduction of these edges induces a substantial distribution shift in the graph topologies, which makes the initially learned product space embeddings no longer a good fit. This causes the Product GCN to underperform relative to the Euclidean GCNs with their respective pretrained embeddings. One conclusion we can draw is that common Euclidean-style embeddings are more robust to distribution shifts of graph topology while product space embeddings, via their learned curvatures, impose a strong inductive bias to tree-like and ring-like structures in the graphs.

## 5. Discussion and Conclusion

We find that performing representation learning in non-Euclidean and mixed-curvature spaces yields notable improvements in distortion and downstream in-distribution edge prediction performance. In all cases, a mixed-curvature representation yields an embedding with lower distortion than a simple Euclidean embedding. However, the exact decomposition of the product space into its mixture of components is highly dependent on the graph topology. Thus, it is beneficial to perform a hyperparameter sweep over the number and types of components, as well as their dimensionalities, when learning a representation for a biological pathway graph. Such a sweep need not be highly time intensive as biological pathway graphs are generally of a modest size.

For the out-of-distribution edge prediction task, the full structure of the graph is not known when the node embeddings are learned because the test set edges from STRING are not included in the training set. This leads to a number of questions about how to further generalize our approach. For example, we may ask how close the full graph structure must be to the observed structure in order to produce node embeddings that are usable for edge prediction via our method. We can also explore whether there are alternative hyperparameter tuning strategies that produce slightly worse distortions or in-distribution edge prediction performance but generalized better to out-of-distribution edge prediction. The Laplacian embedding has the best out-of-distribution performance, consistent with its robust behavior in previous biological graph representation learning benchmarking (Song et al., 2023). Currently, we only assess out-of-distribution edge predictions using STRING physical protein interactions. An evaluation of 45 interaction networks (Wright et al., 2025) could be used to prioritize other networks for evaluating these predictions and understand biases in the interaction networks.

Another future direction would be to consider additional downstream tasks, as edge prediction is not the only useful task for biological pathways that can be improved by non-Euclidean representation learning. We plan to investigate how our pathway embeddings benefit other problems such as node classification, for example, predicting the type (gene, protein, small molecule, metabolite, etc.) of a pathway node.

The lower performance of all models on the out-of-distribution edge prediction setting relative to the in-distribution setting may be partially attributable to biological context. Edges in a pathway reflect a pair of proteins that interact in the context of conducting some specific biological process, potentially only in certain cells or tissue types. The trained GCNs predict edges with that same context. However, the STRING-based evaluation lacks that context. There may be false negative edges in which there is a real PPI that is not relevant to the pathway. Alternatively, there may be false positive edges that are cell- or tissue-specific and not yet identified by the experiments aggregated into STRING.

Non-Euclidean embedding models have not been applied to pathway graphs, perhaps due to a lack of awareness among network biology researchers of their utility in reducing distortion of graph distances and improving downstream predictive performance. We demonstrate that pathway graphs benefit from the incorporation of non-Euclidean geometries into embeddings and prediction models. We encourage researchers to consider making use of these non-standard geometries when learning embeddings and making downstream predictions.

## Supplementary Material

Supplement 1

## Figures and Tables

**Table 1:** General statistics summarizing the five pathway databases.

	PathBank	Reactome	KEGG	HumanCyc	NCI
Number of graphs	875	1723	82	242	212
Number of graphs with single best space	411	884	47	210	144
Number of graphs with multiple best spaces	464	839	35	32	68
Mean number of nodes	63.23	54.36	159.73	104.83	100.95
Mean number of edges	283.56	1091.79	657.39	289.62	531.98
Median number of nodes	53	24	152	73	73
Median number of edges	162	65	532	133	194

## Data Availability

We provide code at https://github.com/mcneela/Mixed-Curvature-Pathways and https://github.com/mcneela/Mixed-Curvature-GCN.

## Footnotes

1 This work extends our workshop paper McNeela et al. (2023).
