# Supplementary material for: Product Manifold Representations for Learning on Biological Pathways
Source: ArXiv. 2025 Feb 4:arXiv:2401.15478v2. Preprint. [Version 2] (PMC11838783)
Supplement: Supplement 1 [file NIHPP2401.15478v2-supplement-1.pdf]

Appendix

A. Supplementary Methods

A.1. Pathway Commons Data

We use the Pathway Commons v12 PathBank pathways provided in this file: https://www.pathwaycommons.org/archives/PC2/v12/PathwayCommons12.pathbank.hgnc.txt.gz

Pathway Commons provides a number of different data formats, including text, SIF, JSON, and BioPAX. We use the text format as it provides the simplest graph representation of the most important interactions in PathBank pathways.

A.2. Initializing GCNs with Embeddings

We save the embeddings learned by the embedding model in a PyTorch .pt file (Paszke et al., 2019). We then load these embeddings and use them to initialize the weights of the embedding layer for the models in the edge prediction task, namely the Euclidean GCN and Product GCN. These embeddings are then further trained via backpropagation during the edge prediction task.

B. Supplementary Tables

**learning rate**	**hyperbolic components**	**Euclidean componentss**	**spherical components**
1e-3	0	0	0
1e-2	1	1	1
1e-1	2	2	2
1	3	3	3

Table 2: Range of values for the pathway embedding hyperparameter sweep. Space dimensions were calculated automatically (to sum to 100) based on the number of components of each space. For example, if there were 2 hyperbolic components, 1 Euclidean component, and 3 spherical components, then there would be floor(100/6) = 16 dimensions assigned to each component.

**learning rate**	**hidden dim**	**# of layers**	**dropout**	**use bias**	**curvature (for Product GCN)**
1e-4	16	2	0.1	true	0.5
1e-3	32	3	0.2	false	1
1e-2	64	4			2

Table 3: Hyperparameters used in the sweep for training the edge prediction GCNs.

C. Supplementary Figures

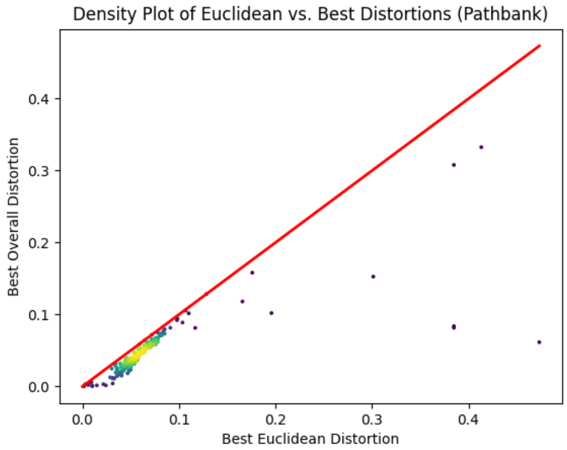

(a) PathBank dataset

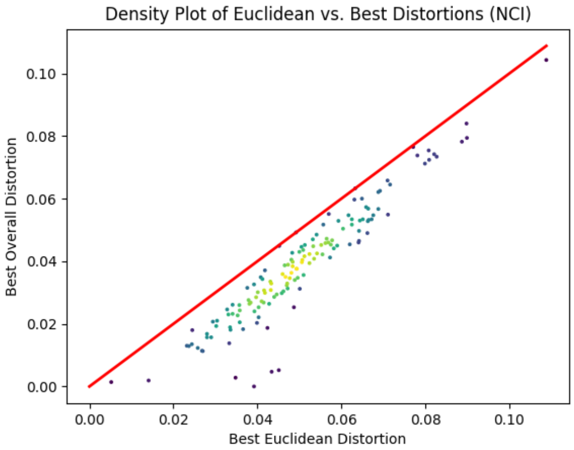

(b) NCI dataset

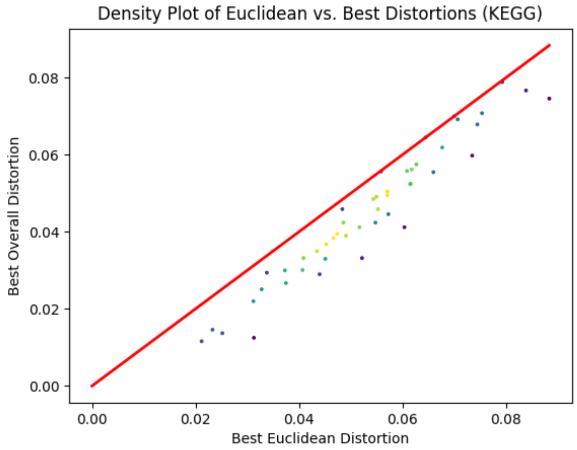

(c) KEGG dataset

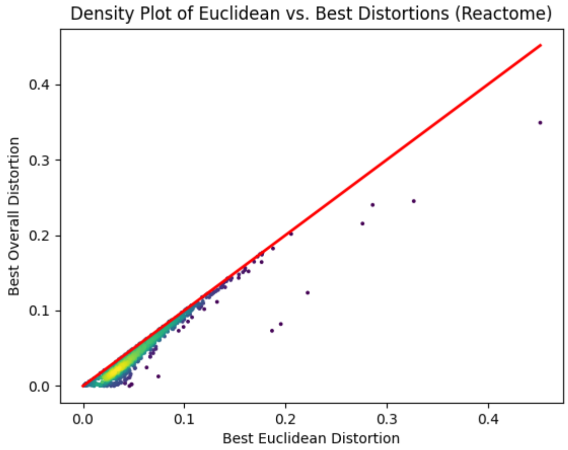

(d) Reactome dataset

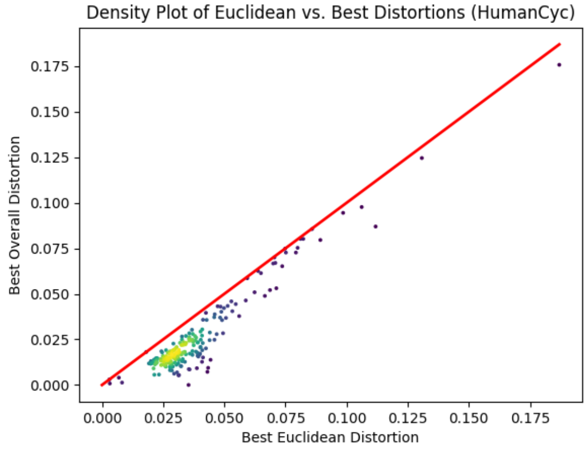

(e) HumanCyc

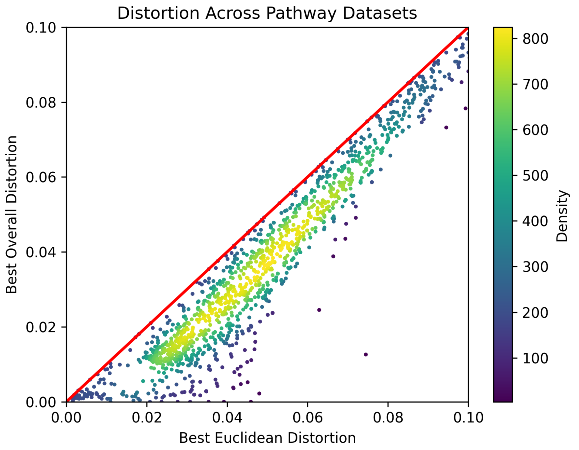

(f) All datasets

Figure 4. Scatterplots of distortion in the Euclidean embedding versus distortion in the mixed-curvature embedding for pathway datasets. Points are colored by local density, with yellow indicating the highest density.

C.1. PathBank

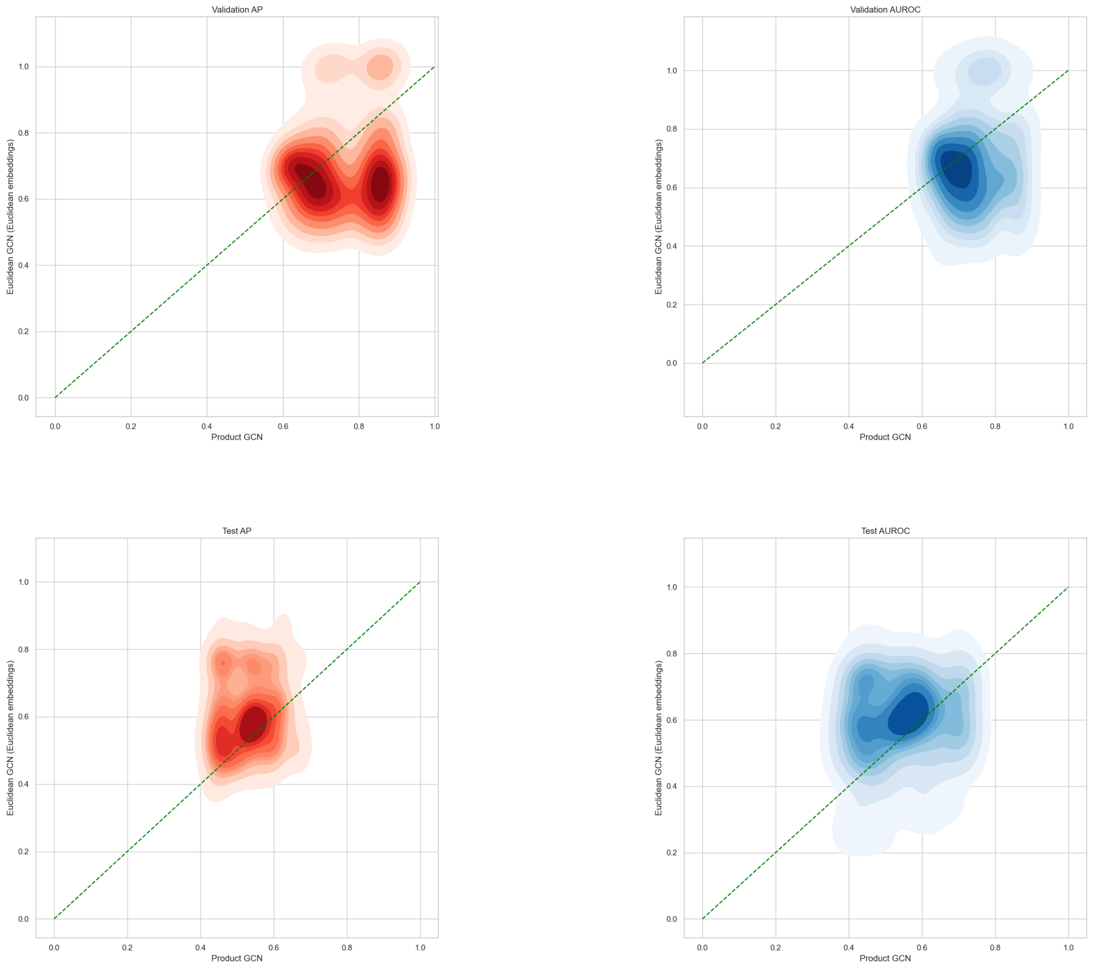

Figure 5. Comparison of Euclidean GCN initialized with pretrained Euclidean embeddings and Product GCN performance on in-distribution validation set and out-of-distribution test set. Each density plot shows one of either AP or AUROC metrics taken across all graphs in the PathBank dataset.

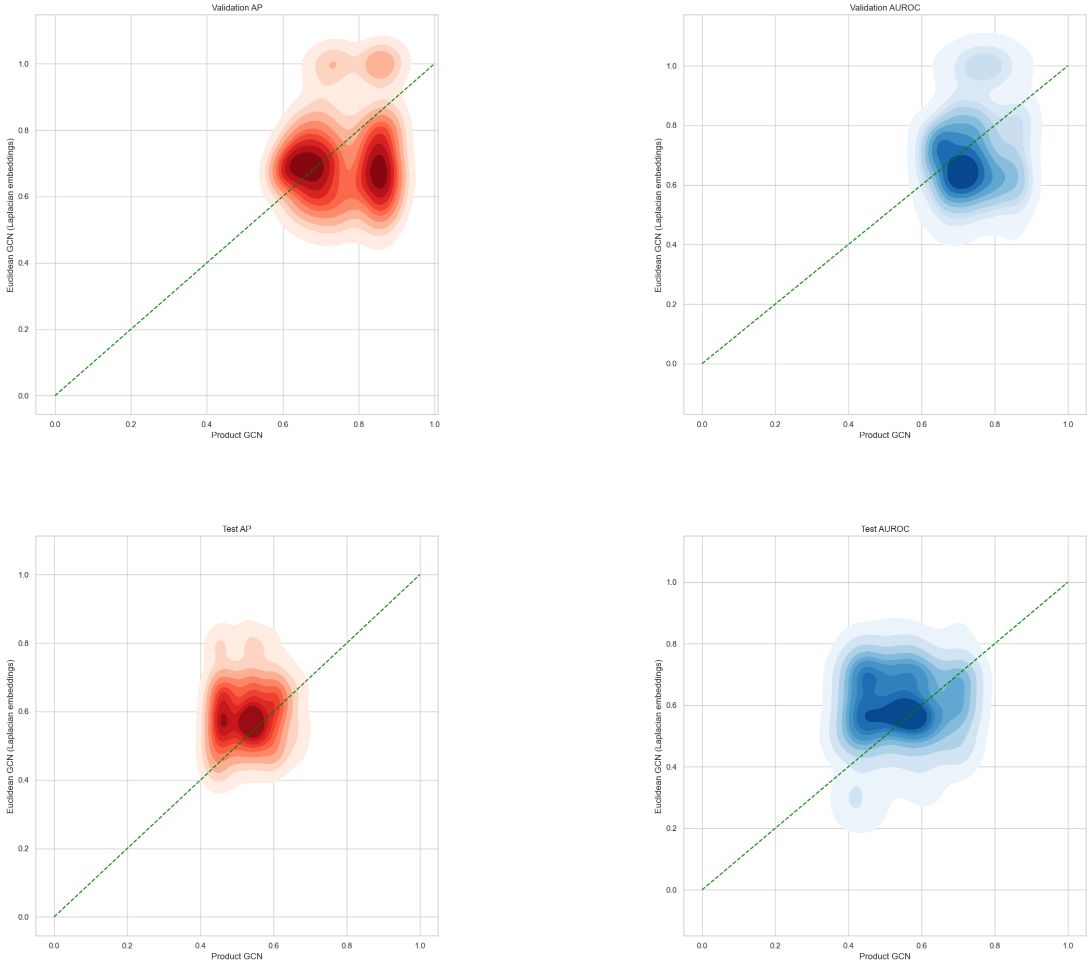

Figure 6. Comparison of Euclidean GCN initialized with pretrained Laplacian embeddings and Product GCN performance on in-distribution validation set and out-of-distribution test set. Each density plot shows one of either AP or AUROC metrics taken across all graphs in the PathBank dataset.

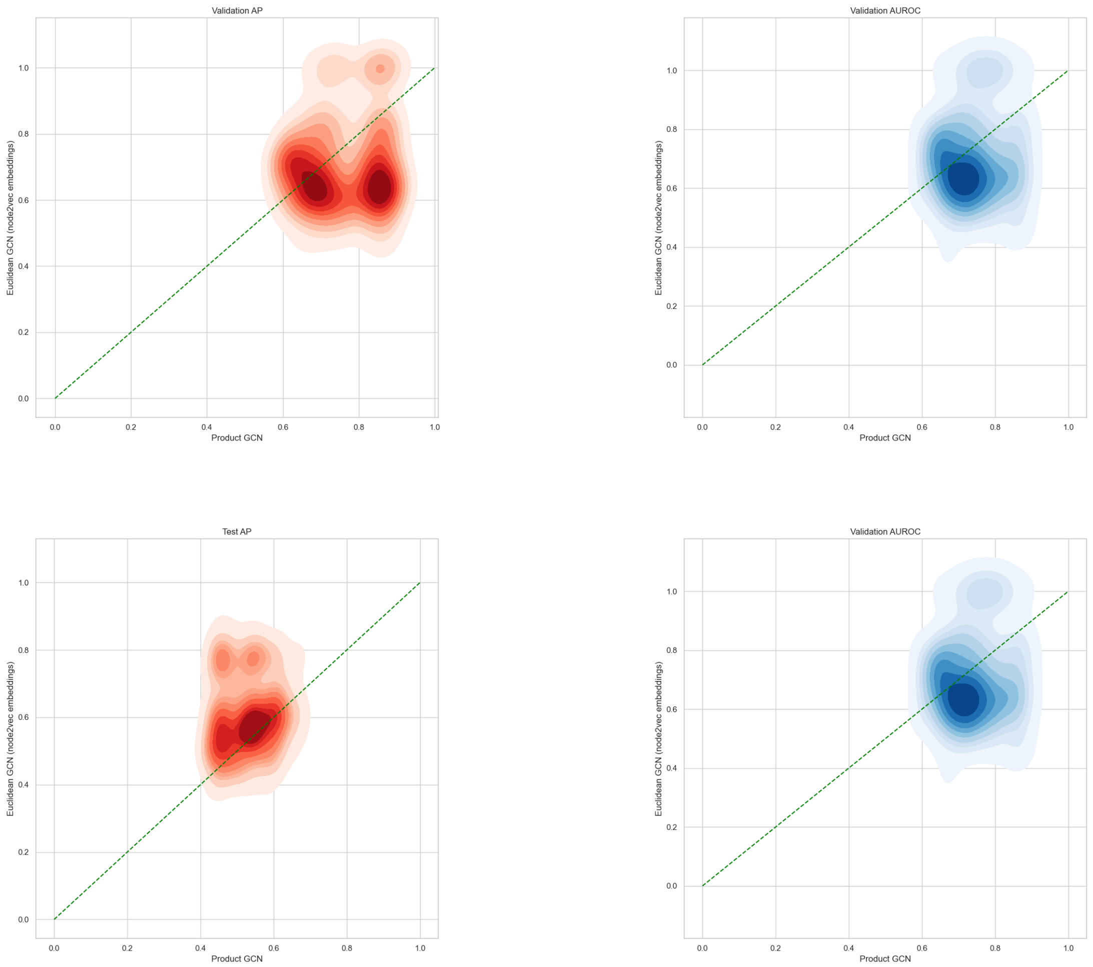

Figure 7. Comparison of Euclidean GCN initialized with pretrained node2vec embeddings and Product GCN performance on in-distribution validation set and out-of-distribution test set. Each density plot shows one of either AP or AUROC metrics taken across all graphs in the PathBank dataset.

C.2. Reactome

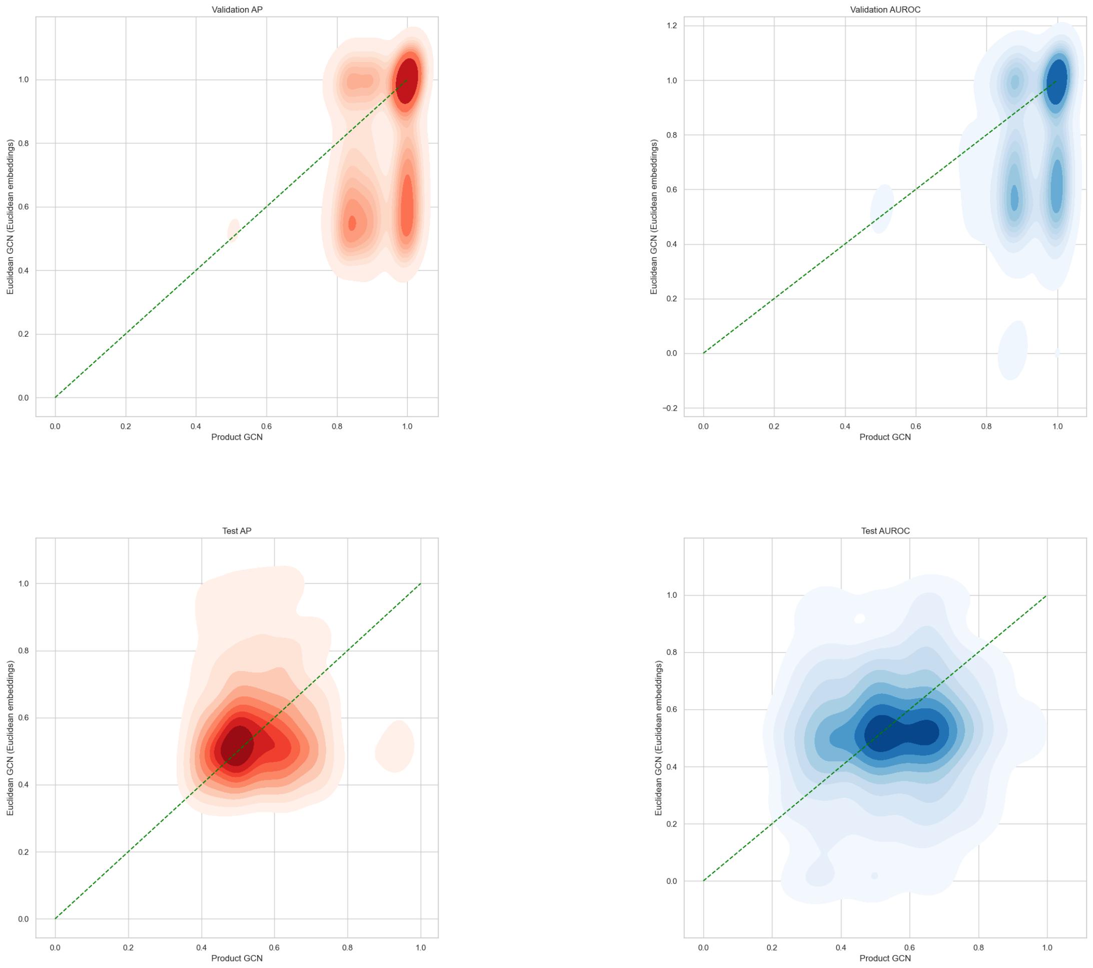

Figure 8. Comparison of Euclidean GCN initialized with pretrained Euclidean embeddings and Product GCN performance on in-distribution validation set and out-of-distribution test set. Each density plot shows one of either AP or AUROC metrics taken across all graphs in the Reactome dataset.

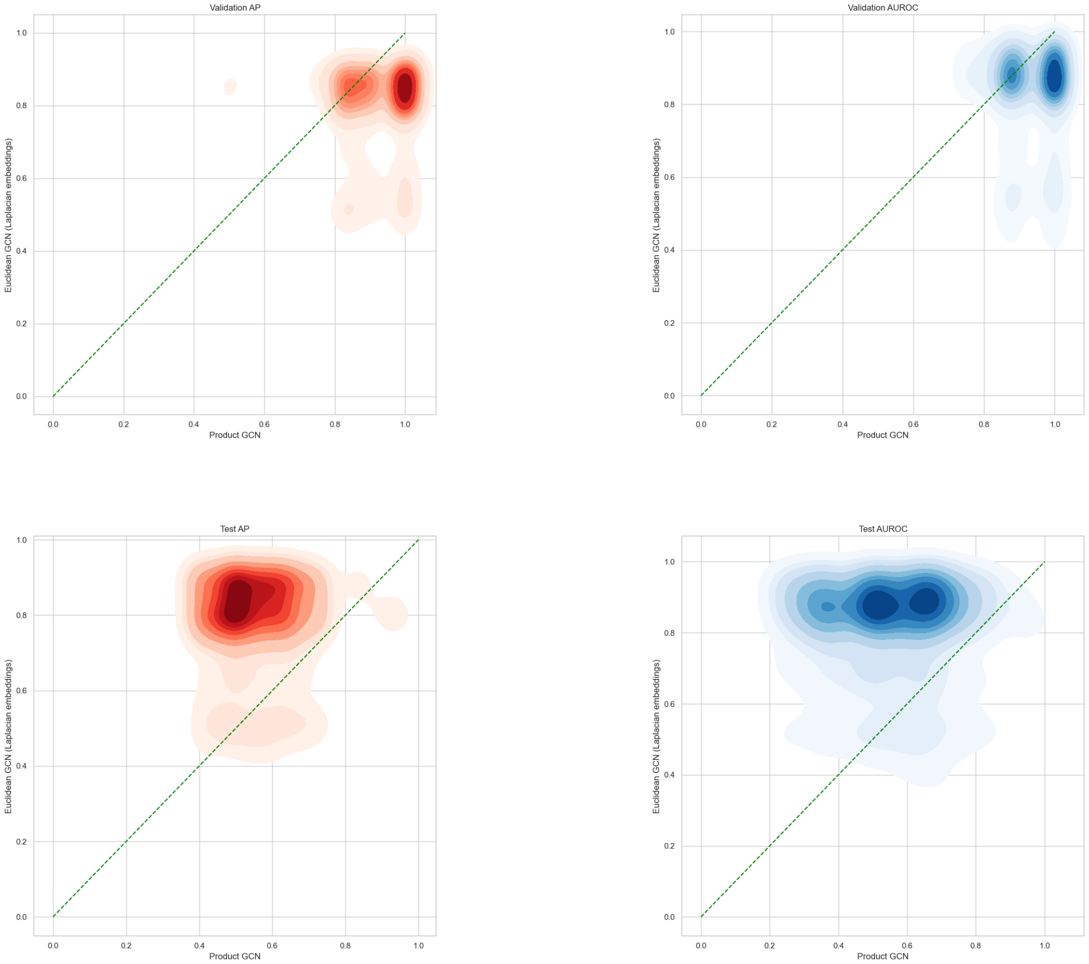

Figure 9. Comparison of Euclidean GCN initialized with pretrained Laplacian embeddings and Product GCN performance on in-distribution validation set and out-of-distribution test set. Each density plot shows one of either AP or AUROC metrics taken across all graphs in the Reactome dataset.

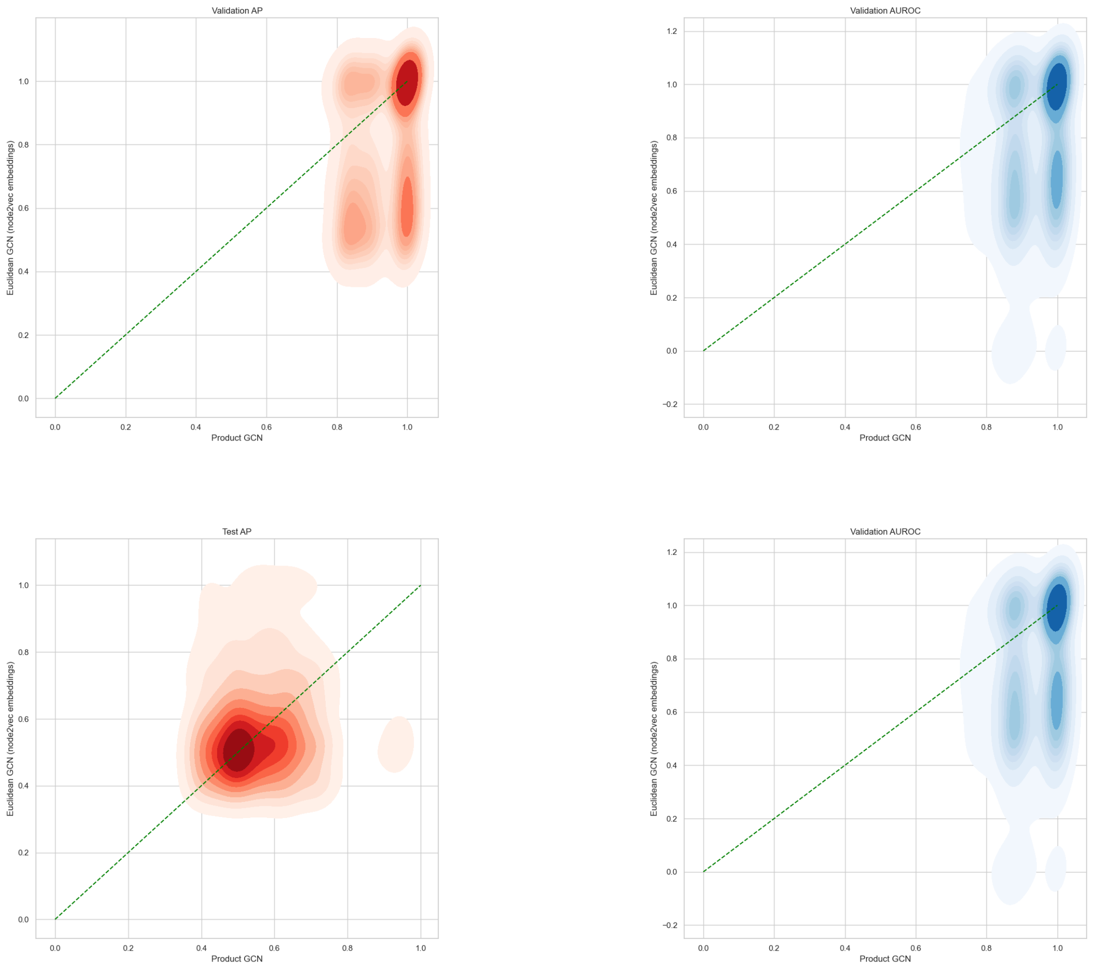

Figure 10. Comparison of Euclidean GCN initialized with pretrained node2vec embeddings and Product GCN performance on in-distribution validation set and out-of-distribution test set. Each density plot shows one of either AP or AUROC metrics taken across all graphs in the Reactome dataset.

C.3. HumanCyc

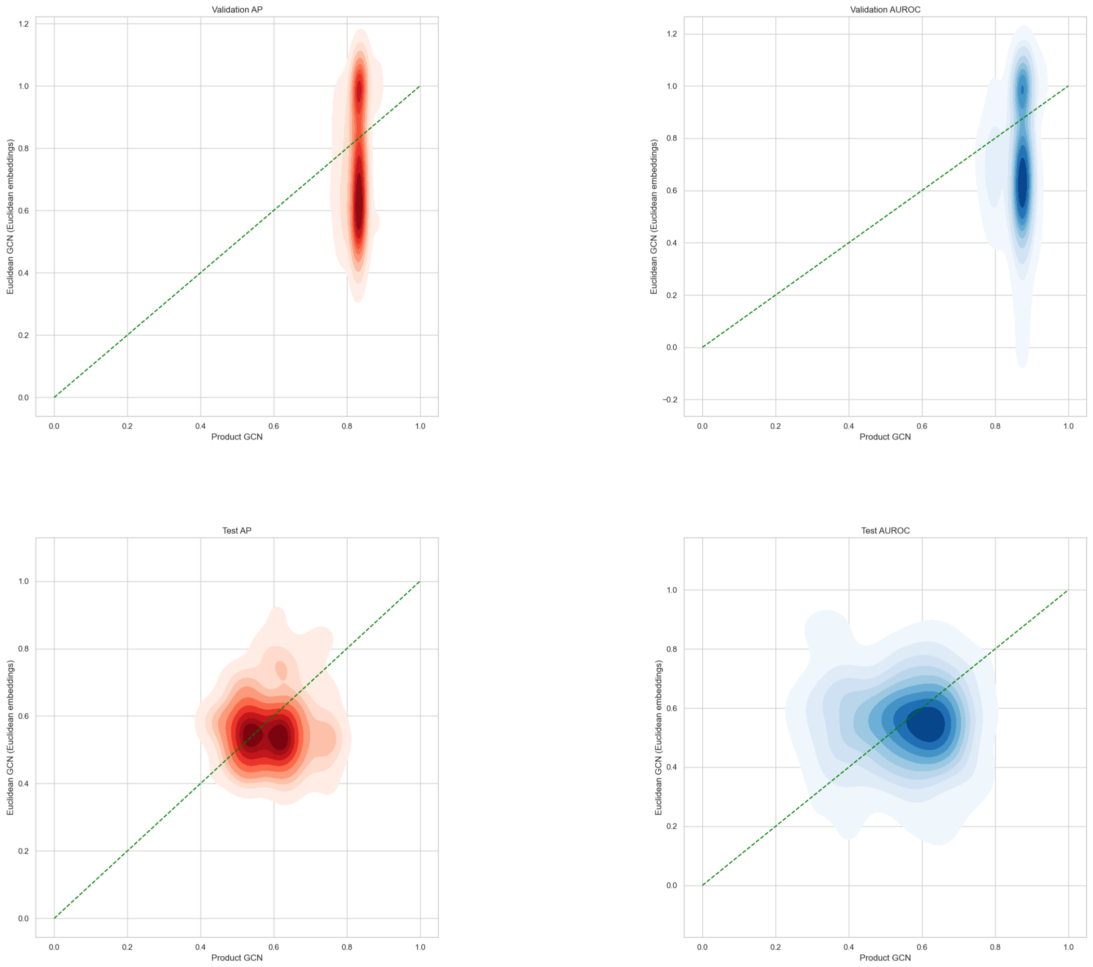

Figure 11. Comparison of Euclidean GCN initialized with pretrained Euclidean embeddings and Product GCN performance on in-distribution validation set and out-of-distribution test set. Each density plot shows one of either AP or AUROC metrics taken across all graphs in the HumanCyc dataset.

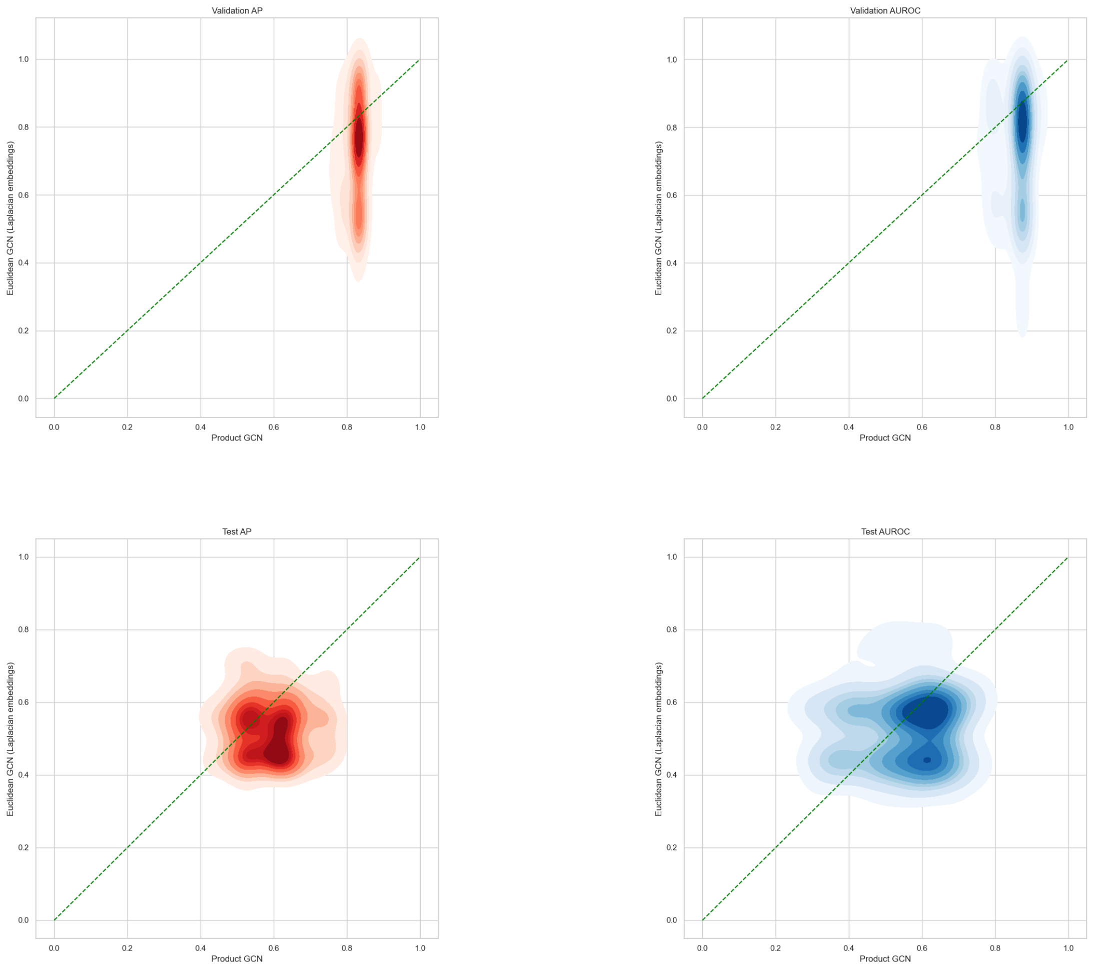

Figure 12. Comparison of Euclidean GCN initialized with pretrained Laplacian embeddings and Product GCN performance on in-distribution validation set and out-of-distribution test set. Each density plot shows one of either AP or AUROC metrics taken across all graphs in the HumanCyc dataset.

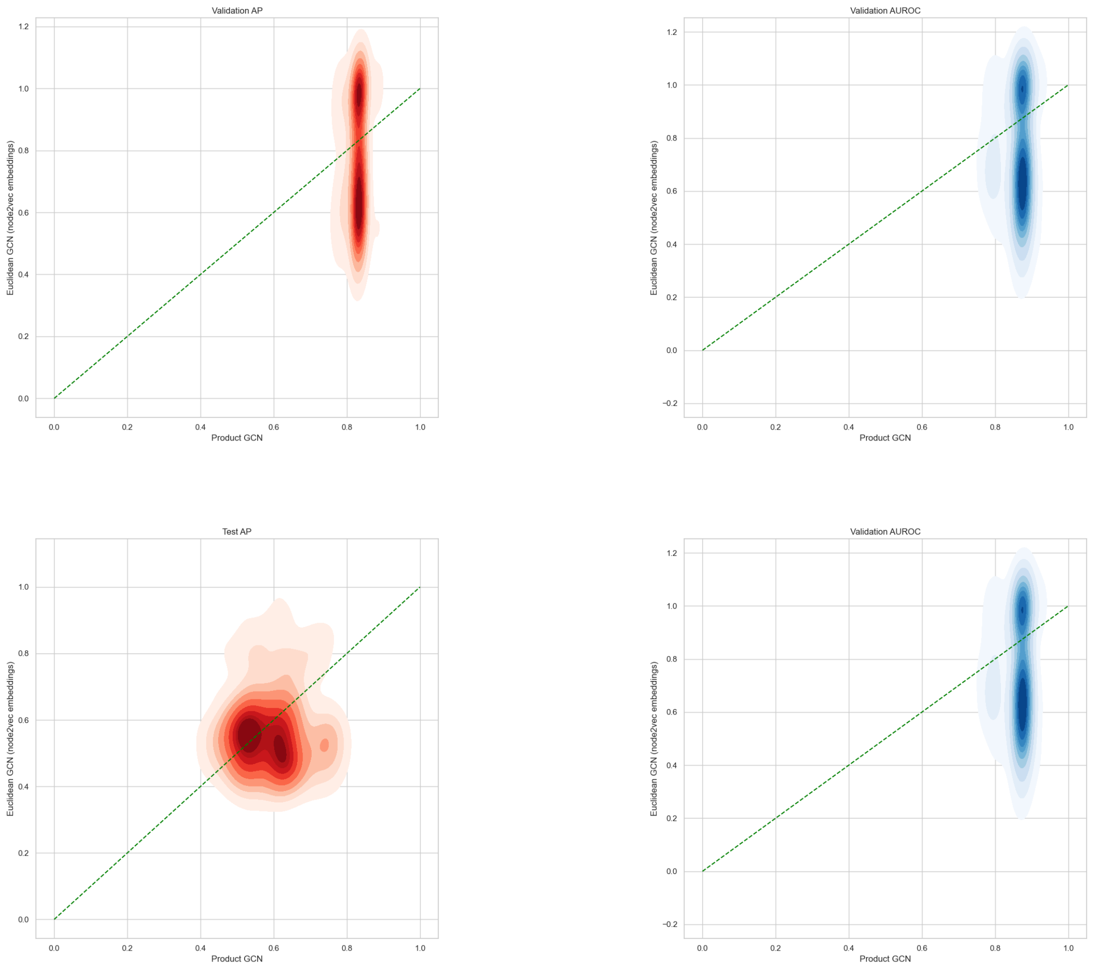

Figure 13. Comparison of Euclidean GCN initialized with pretrained node2vec embeddings and Product GCN performance on in-distribution validation set and out-of-distribution test set. Each density plot shows one of either AP or AUROC metrics taken across all graphs in the HumanCyc dataset.

C.4. NCI

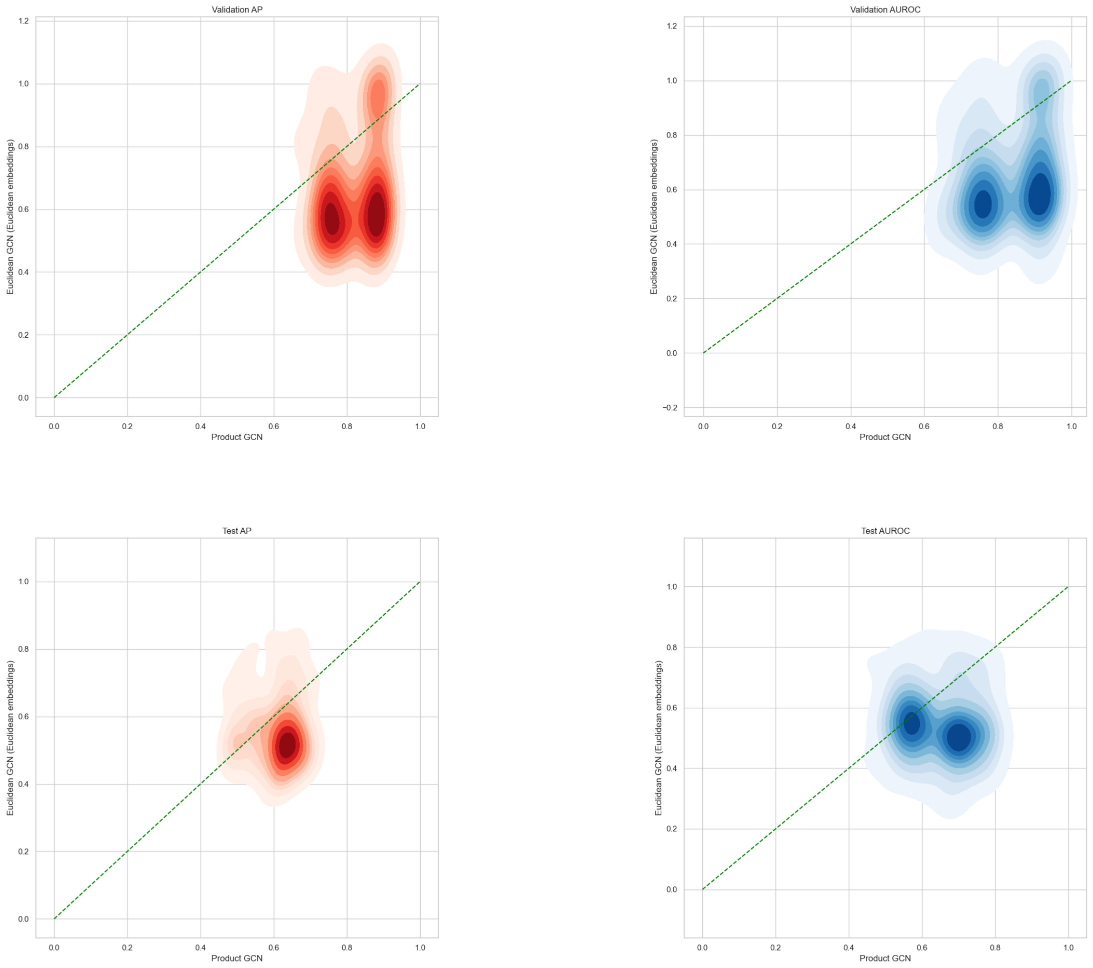

Figure 14. Comparison of Euclidean GCN initialized with pretrained Euclidean embeddings and Product GCN performance on in-distribution validation set and out-of-distribution test set. Each density plot shows one of either AP or AUROC metrics taken across all graphs in the NCI dataset.

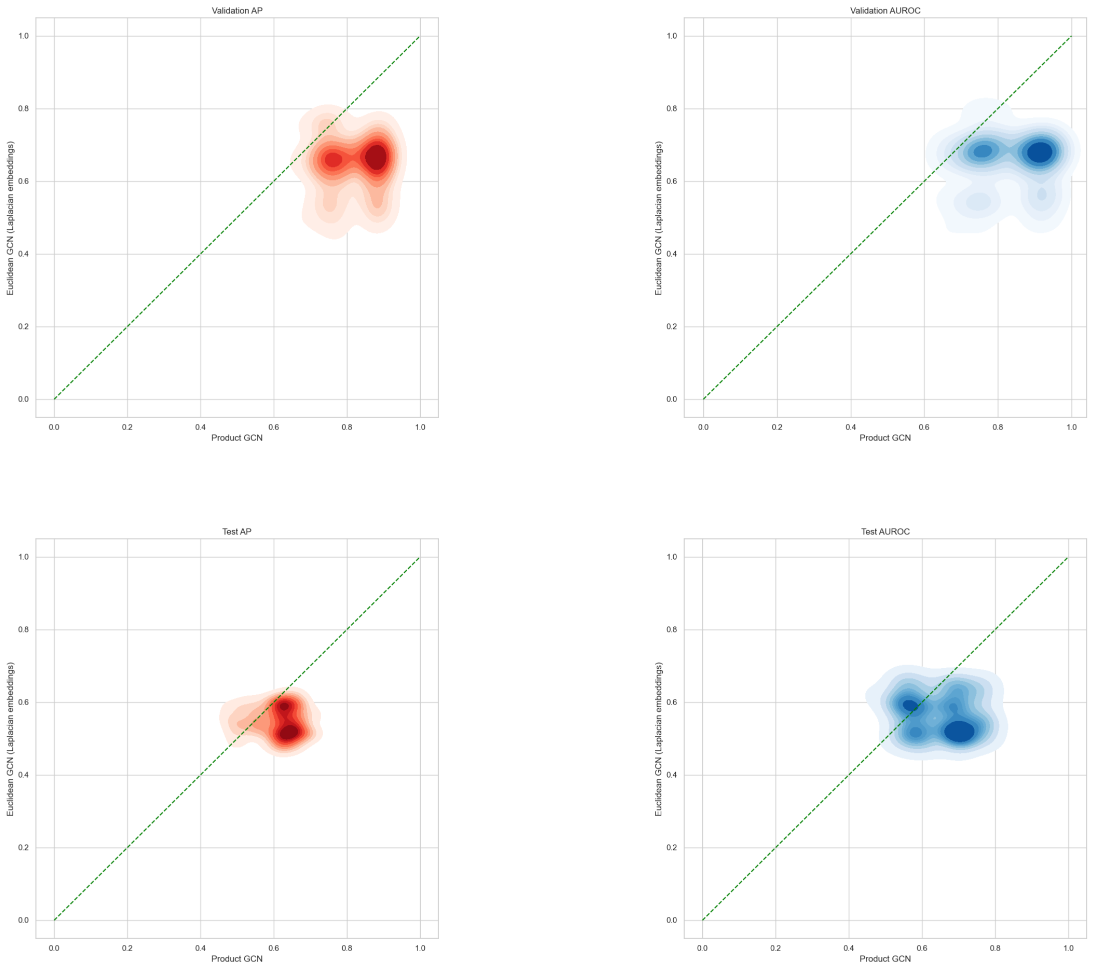

Figure 15. Comparison of Euclidean GCN initialized with pretrained Laplacian embeddings and Product GCN performance on in-distribution validation set and out-of-distribution test set. Each density plot shows one of either AP or AUROC metrics taken across all graphs in the NCI dataset.

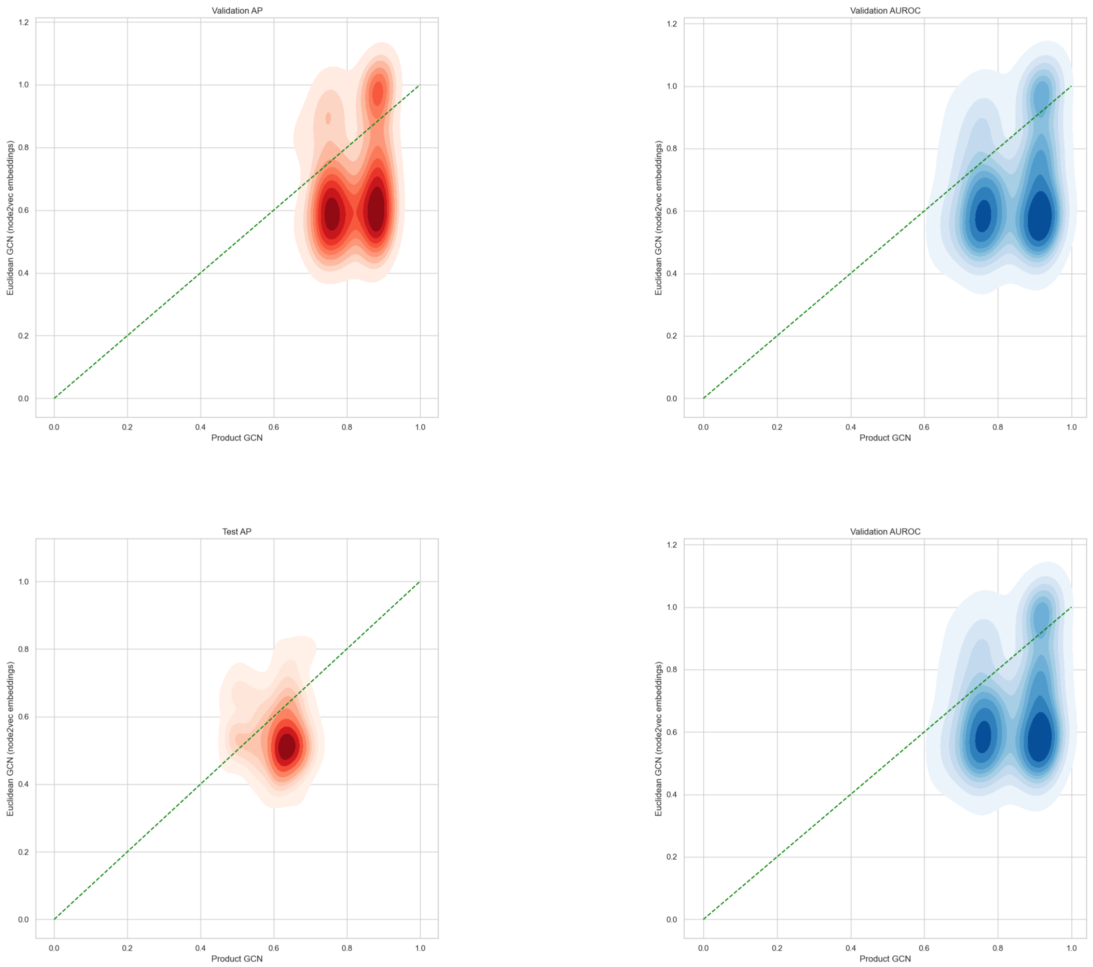

Figure 16. Comparison of Euclidean GCN initialized with pretrained node2vec embeddings and Product GCN performance on in-distribution validation set and out-of-distribution test set. Each density plot shows one of either AP or AUROC metrics taken across all graphs in the NCI dataset.

C.5. KEGG

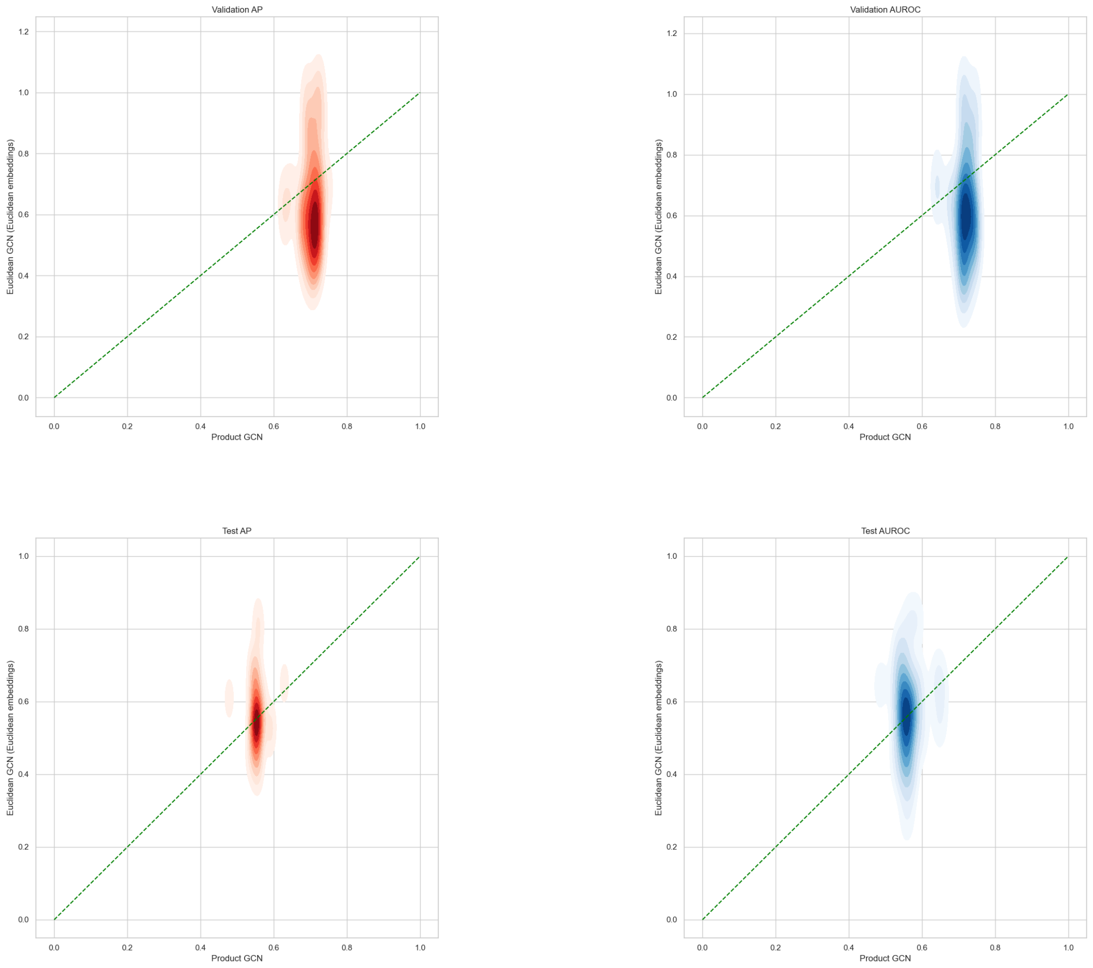

Figure 17. Comparison of Euclidean GCN initialized with pretrained Euclidean embeddings and Product GCN performance on in-distribution validation set and out-of-distribution test set. Each density plot shows one of either AP or AUROC metrics taken across all graphs in the KEGG dataset.

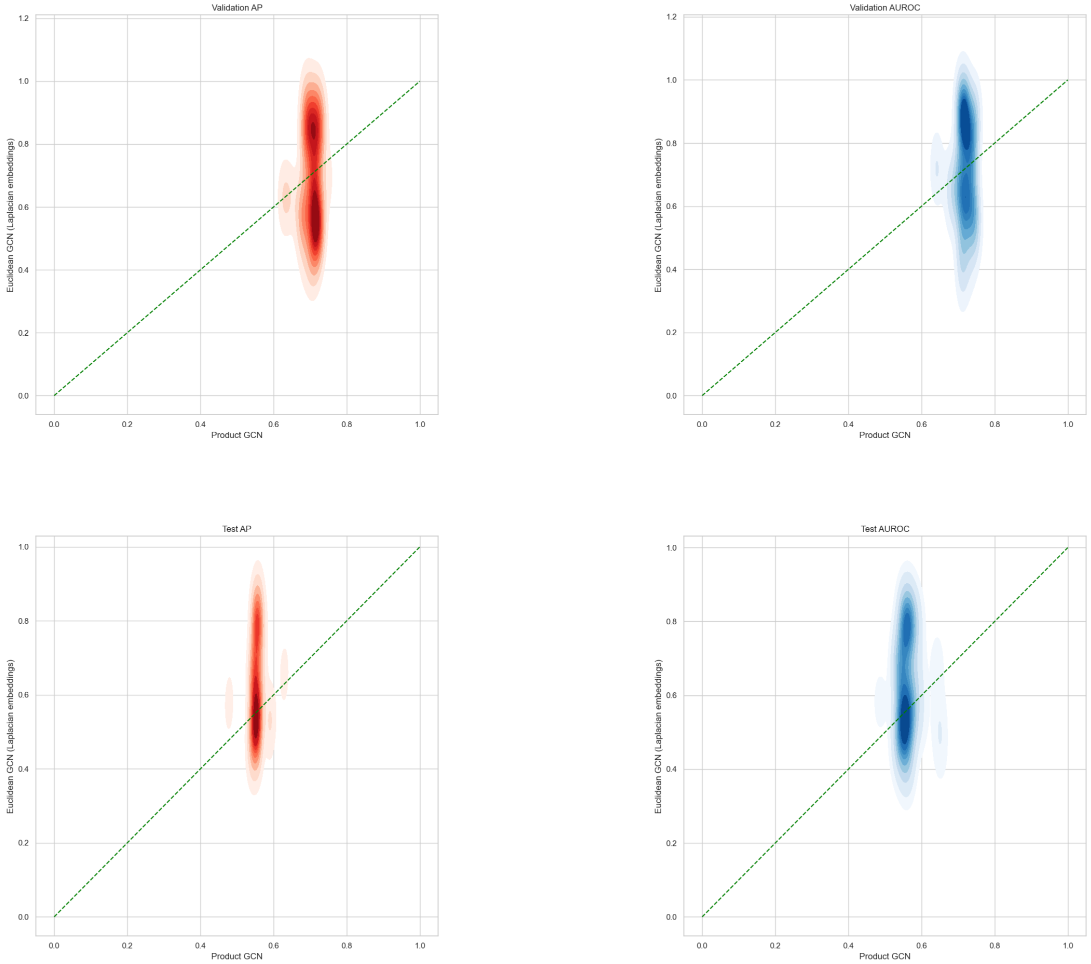

Figure 18. Comparison of Euclidean GCN initialized with pretrained Laplacian embeddings and Product GCN performance on in-distribution validation set and out-of-distribution test set. Each density plot shows one of either AP or AUROC metrics taken across all graphs in the KEGG dataset.

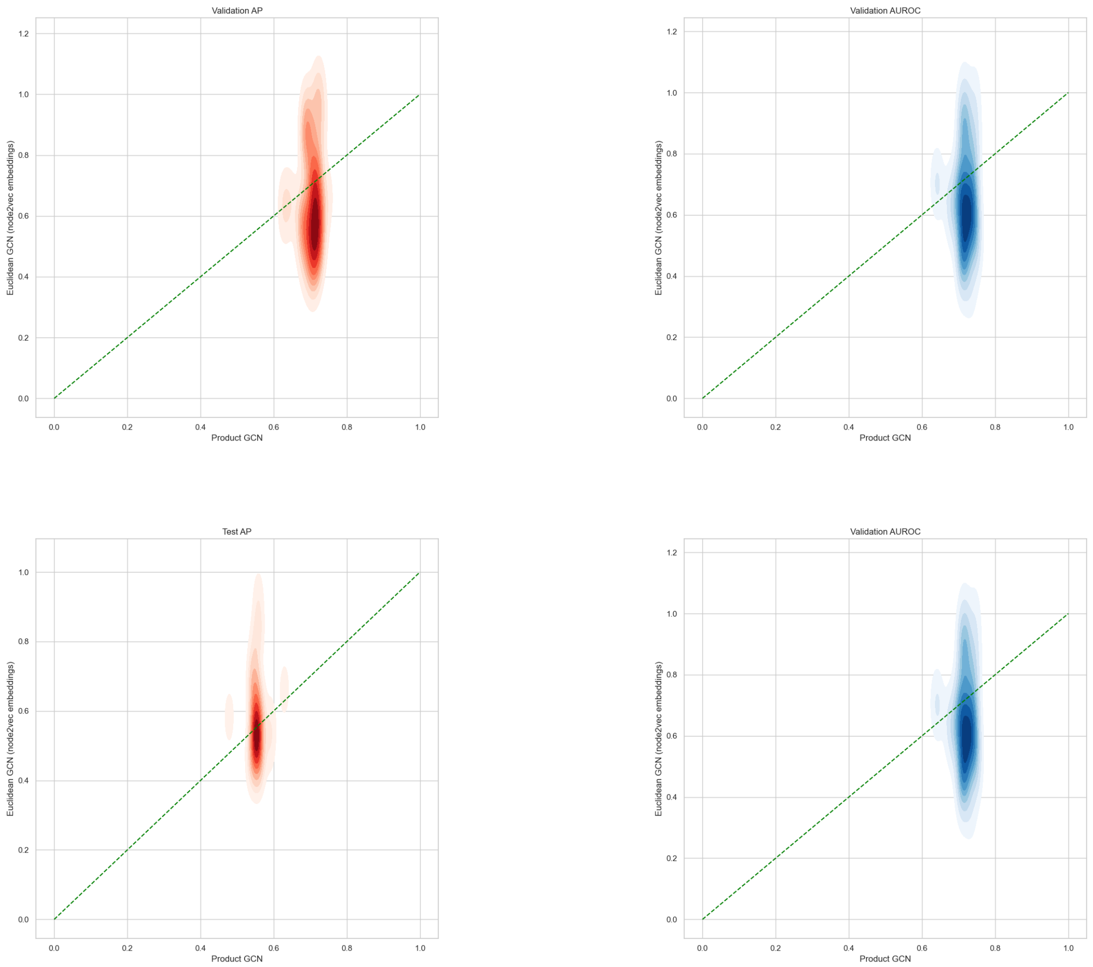

Figure 19. Comparison of Euclidean GCN initialized with pretrained node2vec embeddings and Product GCN performance on in-distribution validation set and out-of-distribution test set. Each density plot shows one of either AP or AUROC metrics taken across all graphs in the KEGG dataset.
